# Policy on the move: active travel to school among 7–16-year-olds in Wales and its links to school policy and socio-demographic factors

**DOI:** 10.1186/s12889-026-26995-0

**Published:** 2026-03-13

**Authors:** Kelly Morgan, Shujun Liu, Safia Ouerghi, Vasiliki Kolovou, John Bradley, Paul Pilkington

**Affiliations:** 1 Centre for Development, Evaluation, Complexity and Implementation in Public Health Improvement (DECIPHer), School of Social Sciences, Cardiff University, Sbarc, Maindy Road, Cardiff, Wales CF24 4HQ UK; 2 Public Health Wales, 2 Capital Quarter, Cardiff, Wales CF10 4BZ UK

**Keywords:** Active travel, School, Intervention, Policy, Health inequalities, Children and adolescents

## Abstract

**Background:**

Active travel to school (ATS) provides an important source of daily physical activity and supports wellbeing. Despite policy interest, national evidence on school promotion of ATS is limited. This study aimed to: (i) estimate the prevalence and socio-demographic variations of ATS among 7–16-year-olds in Wales, UK; (ii) assess school support for ATS; and (iii) examine associations between individual and school-level factors and pupil-reported ATS.

**Methods:**

Survey data were collected from 177,715 pupils (47.1% female) in a nationally representative sample: 51,662 primary school pupils (aged 7–11 years; *N* = 510 schools) and 126,053 secondary school pupils (aged 11–16 years; *N* = 201 schools). Pupil survey data were linked with a School Environment Questionnaire completed by school staff, capturing school-level ATS policies. Multilevel logistic regression models examined associations between pupil characteristics (age, gender, ethnicity) and school characteristics (socioeconomic status, location) and ATS, adjusting for clustering by school.

**Results:**

Overall, 34.1% of pupils reported ATS, with higher rates among primary-aged pupils (*p*<.001). ATS varied significantly by age, gender, and ethnicity. Pupils attending medium- or high-socioeconomic status schools were more likely, and those attending rural schools less likely, to report ATS. Most schools reported at least one ATS policy (primary: 96.7%; secondary: 93.3%), averaging four policies per school, grouped into promotion, infrastructure, training, and partnership initiatives. Among primary school pupils, each additional policy was associated with a 5% increase in odds of ATS (OR = 1.05, 95% CI: 1.01–1.09); the trend among secondary pupils was non-significant (OR = 1.06, 95% CI: 0.99–1.14).

**Conclusions:**

Schools are making considerable efforts to promote ATS, yet national findings reveal persistent inequalities by age, socioeconomic status, and rurality. These results underscore the need for targeted, context-sensitive strategies to ensure equitable access to active travel opportunities across educational settings.

**Supplementary Information:**

The online version contains supplementary material available at 10.1186/s12889-026-26995-0.

## Background

Active travel to school (ATS) is increasingly recognised as a key contributor to children’s overall physical activity levels and broader well-being, potentially providing nearly half of the recommended weekly physical activity [1, 2]. Common modes of travel include walking, cycling, scooting and wheeling (using a mobility aid such as a wheelchair). Active travel is associated with children’s improved physical health outcomes [3], including better weight-related measures [4, 5] and increased cardiovascular fitness [5, 6] and overall well-being [7, 8]. Some evidence suggests active travel interventions may help reduce health inequities among disadvantaged groups of children [9].

The Socio-ecological Model offers a valuable framework for understanding the multifaceted influences on these behaviours [10]. It conceptualises wider factors across individual, social, and environmental domains and highlights the need for addressing multiple levels of influence rather than focusing solely on individual choice.

### Global and United Kingdom trends in ATS

Most studies from high-income countries report that ATS rates decline from primary to secondary school, with walking and cycling more common among younger children than older adolescents [11]. At the individual level, however, evidence regarding demographic variations in ATS is more mixed. Some studies report increasing active school travel with age [12, 13], while others describe U-shaped [14, 15] or curvilinear patterns [16], suggesting that developmental, social, and contextual factors may influence travel behaviour differently across age groups. For instance, greater independence and shifting parental concerns among older children may facilitate ATS, while the transition from primary to secondary school has been associated with a decline in cycling rates, likely due to changes in travel preferences and social norms [17].

Globally research shows similar rates of active travel among girls and boys [18], but in the United Kingdom (UK) a gender gap has been demonstrated. For example, in a UK-based survey of 1089 children aged 6–15, 62% reported daily active travel to school, with 28% of boys reporting cycling compared to 17% of girls [19]. Other studies have also shown higher levels of active travel among boys aged 11-16-years compared to girls [20]. UK studies based within city populations have found notable inequalities, with ethnic minority groups reporting lower levels of active travel to school, compared to white peers [21], though national scale evidence remains limited. Socioeconomic status is another determinant, with inconsistent conclusions: some studies show higher ATS among more deprived groups [22, 23], often citing proximity to schools as a potential rationale [22] while others reveal higher ATS among more affluent groups [24, 25], highlighting walkable neighbourhoods or greater bicycle availability.

Beyond demographics, social factors such as peer behaviours and parental perceptions [26], and features of the built environment, including proximity to school, neighbourhood safety, and street connectivity [27, 28], play a key role in shaping travel choices.

### Schools as key settings for ATS promotion

Schools are widely acknowledged as key settings for promoting health and wellbeing, given their reach into the lives of children and young people during formative years [29]. They shape social norms and daily routines and are well placed to encourage ATS. Global recommendations increasingly advocate for whole-school approaches to physical activity, including active travel initiatives [30].

### Wales policy context and current priorities

While these patterns are observed globally and across the UK, Wales presents a distinctive policy context. Increasing ATS has been identified as a key priority in Wales [31], underpinned by the Active Travel (Wales) Act 2013, which placed a statutory duty on local authorities to promote walking and cycling as modes of everyday travel. Wales has set a national modal‑shift target − 45% of all journeys by sustainable modes by 2040, but no specific targets exist for active school travel [32].

Despite these ambitions, progress has been widely critiqued. The Wales Active Travel Board, in its last two annual reports, highlighted persistent challenges and limited success in meeting targets [33]. Similarly, Audit Wales, in its 2024 report on active travel, raised concerns about the effectiveness of Welsh Government efforts and the need for stronger implementation and accountability [34]. These critiques have intensified the current focus on improving active travel in Wales, making it a particularly important context for research.

To date, interventions aiming to increase ATS have shown mixed results [35, 36]. A recent review [36] of 10 studies focusing on children found that awareness-raising activities, gamification, safety zones, walking or cycling buses, and infrastructure improvements yielded variable outcomes. Interventions focusing solely on knowledge, skills, or attitudes had limited impact [37–39] and infrastructure improvements such as new road layouts, pavement markings and signage yielded minimal effects [40–42]. Walking or cycling bus interventions showed promise [43, 44], but sustainability depends on ongoing funding and engagement. Similarly, gamification interventions [45, 46] were effective among 5-11-year-olds, however, durability of these impacts beyond the intervention period remains unclear.

### Evidence gaps and rationale for the study

Despite policy commitments, there is limited evidence on how schools in Wales actively support ATS and whether these efforts translate into equitable uptake across different groups of young people. Recent calls emphasise the need for context-specific research that explores school-level actions and their impact [22]. Understanding these dynamics is essential for guiding decisions by policymakers, schools, and local authorities on how to allocate resources for maximum impact.

The objectives of this study are to:

1. Estimate the prevalence of ATS among 7–16-year-olds in Wales;
2. Explore how active travel varies by socio-demographic characteristics (age, gender, and ethnicity);
3. Assess how and to what extent schools support ATS; and
4. Examine whether individual characteristics and school-level active travel policies are associated with pupil reported ATS.

## Methods

This cross-sectional study used survey data collected from maintained (i.e., state-funded public) primary and secondary schools^1^ across Wales, UK, via the School Health Research Network (SHRN) [47]. Data collection consisted of two surveys; a Student Health and Well-Being (SHW) survey and a School Environment Questionnaire (SEQ) completed by school staff (e.g., members of the school leadership team) [48]. The SEQ was developed using items drawn from an established international school-health survey [49] and reviewed by public-health specialists to ensure contextual relevance. Surveys were conducted in the Autumn school term, with all maintained secondary schools invited to participate in 2023 and all maintained primary schools invited to participate in 2024.

Consent was obtained through a three-tiered opt-out process. First, schools provided institutional consent by signing and returning a completed research agreement. They then distributed participant information sheets and a copy of the questionnaire to parents/carers, outlining the purpose and content of the study. Parents/carers were given 14-days to opt their child out of participation by informing the school. Finally, children were informed about the survey both in advance and on the day of data collection and were given the opportunity to decline participation.

Of the 1,211 invited primary schools, 510 participated, with 51,662 pupils aged 7–11 taking part (80.6% of the expected cohort of 64,115). School participation rates were higher in urban (48.5%) than in rural (33.6%) areas, and participating schools were evenly distributed across Free School Meal (FSM) tertiles: Low (33.76%), Medium (33.33%), and High (32.91%). For further details regarding the profiles of participating versus non-participating primary schools, please refer to the supplementary material (Table C – Table E).

At the secondary school level, 96% maintained secondary schools in Wales (*n* = 201) participated, with 129,761 Year 7–11 pupils taking part (75% of the expected cohort of 173,015). Restricting the sample to pupils aged 11–16 years resulted in a final analytical sample of 126,053 pupils.

The SHW and SEQ datasets for primary and secondary schools were combined. At the primary school level, data from 48,998 pupils were successfully merged, achieving a linkage rate of 94.84%. However, 2,664 pupils could not be linked to a specific school. Cases where linkage were unsuccessful were due to missing school identifiers in the SHW survey and these unmatched records were excluded from the final analytical sample. At the secondary school level, 93.78% (*n* = 118,214) of the pupils in our target analytical sample (*n* = 126,053 pupils aged 11–16) were successfully matched to their respective institutional records.

### Student survey

#### Demographics

Within the SHW, pupils were presented with a series of demographic questions. These included school year (response options ranged from Year 3 (age 7-8-years) to Year 11 (15-16-years)), gender (response options: “Boy”, “Girl”, “I don’t want to answer”, and “Neither word describes me”) and ethnicity. As noted elsewhere [48], ethnicities are reported as aggregate, higher‑order categories. These categories were applied during post‑collection coding rather than presented directly to participants. Ethnicity responses were coded into the following groups: ‘White’, ‘Black’, ‘Asian’, ‘Mixed or multiple’, ‘Other’, and ‘I do not want to answer’. In contrast, the primary school survey presented these aggregated categories directly to pupils as response options, with the addition of ‘Gypsy, Roma or Traveller’^2^ as a distinct category.

#### Active travel

Pupils also reported their usual mode of transport to school by responding to the question: “On a typical day, what is the main part of your journey to school made by?” with response options including walking, bicycle, public transport (bus, train, tram, underground, or boat), car, motorcycle or moped, other means, and “I don’t want to answer.” Travel modes were dichotomized as active (encompassing “walking” and “bicycle,” coded as 1) or non-active (including “bus, train, tram, underground, or boat,” “car, motorcycle, or moped,” and “other means,” coded as 0).

### School Environment Questionnaire

#### Total number of active travel policies

The school leadership team was asked to report on the ways in which they promoted active travel by selecting one or more of eight response options. These included identifying and promoting safe walking and cycling routes, providing secure covered storage for bicycles and scooters, promoting the use of helmets, organising walking initiatives (e.g., Walk to School Week), offering cycling proficiency training, providing dedicated pedestrian or cyclist entrances, and collaborating with police or Police Community Support Officers (PCSOs)^3^to address local transport and community safety, and other. Schools could select all applicable options. Those who reported yes to any existing policy were coded 1 and all alternative responses a 0. A sum score was created to provide an overall policy count for each school.

#### Type of active travel policies

To assess the nature of policies being implemented, active travel response items were further grouped into four travel policy types – (i) promotion (i.e., identifying and promoting safe walking and cycling routes, promoting the use of helmets, and organising walking initiatives), (ii) infrastructure (i.e., providing secure covered storage for bicycles and scooters, and providing dedicated pedestrian or cyclist entrances), (iii) training (i.e., offering cycling proficiency training), and (iv) partnership (i.e., collaborating with police or PCSOs to address local transport and community safety). Each of these were treated as separate binary variables (coded 1 if at least one policy within that category was selected and 0 if not).

### School-level variables

The following data were obtained from Stats Wales for each school and linked to the SEQ dataset.

#### Socioeconomic status

The percentage of pupils eligible for free school meals (%FSM) was used to indicate the school-level socioeconomic status. FSM entitlement at the individual level is a robust indicator of socioeconomic disadvantage in England and Wales. Aggregated at the school level, %FSM provides a continuous measure of school-level socioeconomic composition. Data on FSM eligibility were obtained from Stats Wales [50] and linked to the SEQ dataset. Subsequently, %FSM was recoded into three categorical variables based on tertiles of the distribution, with lower %FSM indicating higher school-level socioeconomic status.

#### Location

Using the Rural Urban Classification (RUC) [50], the geographic location of each school was classified as rural (coded as 0) or urban (coded as 1).

### Analyses

Cross-tabulations were utilised to examine the distribution of active travel across pupils’ demographic characteristics. To investigate the association between school travel policies and pupils’ active travel behaviours, school- and pupil-level datasets were linked, and multilevel logistic regression was employed to account for the hierarchical structure of pupils nested within schools. School travel policies were modelled using two analytical approaches. After estimating models including pupil-level predictors only (i.e., Model 1a, Model 2a in Tables 4 and 5), the number of school travel policies was first entered as a continuous variable to assess whether a higher number of policies was associated with increased odds of active travel among pupils (i.e., Model 1b, Model 2b). Subsequently, four types of travel policies (i.e., promotion, infrastructure, training, and partnership) were included as separate binary variables to evaluate whether specific policy types were differentially associated with pupils’ active travel behaviour (i.e., Model 1c, Model 2c). Control variables included pupils’ gender, grade, ethnicity, as well as school-level factors such as the percentage of pupils eligible for free school meals (%FSM) and school location. Odds ratios were reported to quantify the strength of associations, and intra-class correlations (ICCs) were estimated to present how much of the variability for pupils’ active travel could be accounted for by the school level. Model fit was assessed using log-likelihood, Akaike information criterion (AIC), and Bayesian information criterion (BIC) values.

As the missing data rate for most variables was below 10% (see Supplementary file1 Tables A and B) for both primary and secondary samples, listwise deletion was applied to address missing values. All analyses were conducted using Stata, 18.0.

## Results

### Prevalence of active travel to school

The full sample size included 177,715 pupils (47.12% female). Of these, 51,662 were primary school aged pupils (7-11-year-olds) and 126,053 secondary school aged (11-16-year-olds) pupils. Tables 1 and 2 display descriptive characteristics of the study sample.

**Table 1:** Prevalence of active travel to school across demographic features among 7-11-years-olds in Wales

	Active Travel	Non-Active Travel	Total
Gender			
Boys	9,566 (39.93%)	14,390 (60.07%)	23,956 (100.00%)
Girls	9,543 (39.51%)	14,610 (60.49%)	24,153 (100.00%)
Other gender identity	144 (40.45%)	212 (59.55%)	356 (100.00%)
	*Chi* ^*2*^ *(2) = 0.968*	*p* = .616	
Year group			
Year 3	4,244 (39.89%)	6,395 (60.11%)	10,639 (100.00%)
Year 4	4,800 (39.44%)	7,370 (60.56%)	12,170 (100.00%)
Year 5	4,961 (38.68%)	7,864 (61.32%)	12,825 (100.00%)
Year 6	5,517 (40.94%)	7,959 (59.06%)	13,476 (100.00%)
	*Chi* ^*2*^ *(3) = 14.6384*	*p* = .002	
Ethnicity			
White	13,712 (38.29%)	22,095 (61.71%)	35,807 (100.00%)
Black	867 (51.85%)	805 (48.15%)	1,672 (100.00%)
Asian	848 (48.18%)	912 (51.82%)	1,760 (100.00%)
Mixed or multiples	1,039 (43.97%)	1,324 (56.03%)	2,363 (100.00%)
Gypsy, roma or traveller	81 (34.76%)	152 (65.24%)	233 (100.00%)
Other	218 (46.19%)	254 (53.81%)	472 (100.00%)
	*Chi* ^*2*^ *(5) = 214.327*	*p* = .000	

**Table 2:** Prevalence of active travel to school across demographic features among 11-16-years-olds in Wales

	Active Travel	Non-Active Travel	Total
Gender			
Boys	21,277 (35.34%)	38,935 (64.66%)	60,212 (100.00%)
Girls	18,426 (30.93%)	41,154 (69.07%)	59,580 (100.00%)
Other gender identity	530 (32.94%)	1,079 (67.06%)	1,609 (100.00%)
	*Chi* ^*2*^ *(2) = 262.92*	*p* = .000	
Year group			
Year 7	9,105 (34.38%)	17,378 (65.62%)	26,483 (100.00%)
Year 8	8,363 (32.14%)	17,660 (67.86%)	26,023 (100.00%)
Year 9	8,434 (33.26%)	16,920 (66.74%)	25,354 (100.00%)
Year 10	7,575 (32.98%)	15,390 (67.02%)	22,965 (100.00%)
Year 11	7,069 (32.85%)	14,448 (67.15%)	21,517 (100.00%)
	*Chi* ^*2*^ *(4) = 31.44*	*p* = .000	
Ethnicity			
White	33,572 (32.70%)	69,105 (67.30%)	102,677 (100.00%)
Black	868 (36.83%)	1,489 (63.17%)	2,357 (100.00%)
Asian	1,759 (32.88%)	3,591 (67.12%)	5,350 (100.00%)
Mixed or multiples	1,516 (38.48%)	2,424 (61.52%)	3,940 (100.00%)
Other	954 (37.77%)	1,572 (62.23%)	2,526 (100.00%)
	*Chi* ^*2*^ *(4) = 98.74*	*p* = .000	

Among primary aged pupils, almost two fifths (37.79%) reported ATS, with 4.94% revealing missing data. The majority reported active travel through walking (34.43%) as opposed to cycling (3.35%). Among secondary aged pupils (11-16-years), under one third reported ATS (32.17%), with 64.89% reporting non-active travel and 2.94% presenting missing data. The most common mode of active travel among secondary aged pupils was also walking (30.81%).

### Active travel and socio-demographic characteristics

Among 7-11-year-olds (see Table 1), similar rates of ATS were found between boys and girls (39.93% vs. 39.51%, *p* = .616). Comparisons across school year groups revealed the highest levels of ATS among Year 6 pupils (40.94%, χ²(3) = 14.64, *p* = .002 v). Prevalence rates were found to differ according to ethnic backgrounds (χ²(5) = 214.33, *p* < .001), with the highest rates among pupils of Black ethnicity (51.85%) and lowest among pupils reporting a Gypsy, Roma or Traveller background (34.76%).

Among 11-16-year-olds (see Table 2), the prevalence of ATS was highest among boys (35.34%), compared to girls (30.93%) and those reporting other gender identity (32.94%) (χ²(2) = 262.92, *p* < .001). Pupils in Year 7 (aged 11–12 years) reported the highest prevalence of ATS (34.38%), with lower rates observed in older year groups (ranging from 32.14% to 33.26%). While differences between year groups were small, the association was statistically significant (χ²(4) = 31.44, *p* < .001). Differences in prevalence rates were observed among ethnic groupings (χ²(4) = 98.74, *p* < .001). Pupils from mixed or multiple ethnic backgrounds reported the highest ATS prevalence rates (38.48%) while White pupils reported the lowest rates (32.7%).

### School-level support for active travel

The majority of primary (96.67%) and secondary (93.26%) schools reported at least one school-level policy to support active travel (see Table 3), with four policies per school on average. In primary schools (see Table 3), the most commonly reported active travel policies were providing secure covered storage for bicycles and scooters (76.91%), arranging cycling proficiency training (76.13%), and ongoing walking promotions such as *Walk to School Week* (72.02%). By comparison, secure covered storage for bicycles and scooters (65.28%) and collaboration with police/PCSOs (53.37%) were the most common policies by secondary schools.

**Table 3:** Distribution of active travel policy by primary and secondary school

School Policy	Primary schools (*N* = 511)		Secondary schools (*N* = 193)	
	Yes	No	Yes	No
Identify and promote safe walking and cycling routes	258 (50.49%)	253 (49.51%)	83 (43.01%)	110 (56.99%)
Secure covered storage for bicycles and scooters	393 (76.91%)	118 (23.09%)	126 (65.28%)	67 (34.72%)
Promotion of helmets for cyclists	194 (37.96%)	317 (62.04%)	43 (22.28%)	150 (77.72%)
Walking promotions, e.g., “Walk to School Week”	368 (72.02%)	143 (27.98%)	53 (27.46%)	140 (72.54%)
Cycling proficiency training	389 (76.13%)	122 (23.87%)	23 (11.92%)	170 (88.08%)
Pedestrian/cyclist entrances	174 (34.05%)	337 (65.95%)	76 (39.38%)	117 (60.62%)
Collaboration with police/PCSOs to address community/transport safety	306 (59.88%)	205 (40.12%)	103 (53.37%)	90 (46.63%)
Other	35 (6.85%)	476 (93.15%)	6 (3.11%)	187 (96.89%)
None of the above	17 (3.33%)	494 (96.67%)	13 (6.74%)	180 (93.26%)
Don’t know			6 (3.11%)	187 (96.89%)

529 primary schools participated in the survey, but 18 schools did not provide responses to active travel. 214 secondary schools participated, with 21 as missing values

### Associations between socio-demographic characteristics and active travel

Based on model comparisons using AIC, BIC, and log-likelihood, model fit improved significantly following the inclusion of school-level variables (see Tables 4 and 5). Consequently, the results from the full models are reported. As shown in Model 1b (Table 4), among primary aged pupils, there were no differences in the odds of ATS when comparing reports across genders and year groups. Compared with pupils of White ethnicity, those from a Black background had 1.39 times higher odds of reporting active travel (OR = 1.39, 95%CI: 1.23–1.56), whereas pupils from Gypsy, Roma, or Traveller backgrounds had 42% lower odds (OR = 0.58, 95%CI: 0.43–0.80). The ICCs score suggests that 12% of the variance in the likelihood of ATS is attributed to differences between schools, while the remaining 88% is due to individual-level factors or residual variation.

**Table 4:** Multilevel logistic regression predicting active travel to school among 7-11-years olds

	Model 1a			Model 1b			Model 1c		
	O.R.	95% CI	p	O.R.	95% CI	p	O.R.	95% CI	p
Individual level									
Gender (Boy)									
Girl	.99	.95 - 1.03	.627	.98	.94 - 1.02	.328	.98	.94 - 1.02	.328
Other identity	1.05	.82 - 1.35	.703	1.13	.86 - 1.49	.365	1.13	.86 - 1.49	.366
Year (3)									
4	.96	.90 - 1.02	.201	.97	.91 - 1.04	.403	.97	.91 -1.04	.408
5	.98	.92 - 1.04	.507	.97	.91 - 1.04	.408	.97	.91 - 1.04	.415
6	1.05	.99 - 1.11	.139	1.05	.99 - 1.12	.129	1.05	.99 - 1.12	.125
Ethnicity (White)									
Mixed	1.09	.99 - 1.19	.071	1.07	.98 - 1.18	.144	1.08	.98 - 1.18	.142
Asian	1.09	.98 - 1.22	.116	1.07	.96 - 1.21	.224	1.08	.96 - 1.21	.210
Black	1.39	1.25 - 1.57	<.001	1.39	1.23 - 1.56	<.001	1.39	1.23 - 1.56	<.001
Gypsy, Roma or Traveller	.64	.48 - .86	.003	.58	.43 - .80	.001	.58	.43 - .80	.001
Other	1.13	.92 - 1.37	.242	1.06	.86 - 1.31	.601	1.06	.86 - 1.31	.607
School level									
Socioeconomic status (Low)									
Medium	-	-	-	1.18	1.00 - 1.39	.052	1.18	1.00 - 1.38	.068
High	-	-	-	2.22	1.87 - 2.64	<.001	2.18	1.83 - 2.59	<.001
Rurality (Urban)									
Rural	-	-	-	.71	.61 - .83	<.001	.71	.61-.83	<.001
Number of policies	-	-	-	1.05	1.01 - 1.09	.015	-	-	-
Policy contents									
Promotional	-	-	-	-	-	-	1.20	.97 - 1.47	.087
Infrastructure	-	-	-	-	-	-	1.20	.99 - 1.46	.066
Training	-	-	-	-	-	-	.93	.78 - 1.11	.410
Partnerships	-	-	-	-	-	-	.92	.80 - 1.06	.262
Variance (Level 2)	.65	.56 -.76	-	.44	.37 - .51	-	.44	.37 - .51	-
Log likelihood	-26227.76			-22915.08			-22914.29		
AIC/BIC	52479.53 / 52583.26			45862.16 / 45998.18			45866.59 / 46028.11		
ICC – Constant only	0.17								
ICC (Level 1)	0.17								
ICC (Level 1 & 2)	0.12								

N = 41,938; OR = odds ratio; ICC = intra-class correlations. Level 1 refers to pupil-level variables and Level 2 refers to school-level variables. Model 1a includes pupil-level predictors only. Model 1b additionally includes school-level predictors, with a focus on the number of policies. Model 1c includes school-level predictors, focusing on policy content

**Table 5:** Multilevel logistic regression predicting active travel to school among 11-16-years olds

	Model 2a			Model 2b			Model 2c		
	O.*R*.	95% CI	*p*	O.*R*.	95% CI	*p*	O.*R*.	95% CI	*p*
Individual level									
Gender (Boy)									
Girl	0.82	0.80 − 0.84	< 0.001	0.82	0.80 − 0.84	< 0.001	0.82	0.80 − 0.84	< 0.001
Other identity	0.89	0.79 − 1.00	0.044	0.90	0.80–1.02	0.114	0.90	0.80–1.02	0.114
Year (7)									
8	0.90	0.87 − 0.94	< 0.001	0.90	0.87 − 0.94	< 0.001	0.90	0.87 − 0.94	< 0.001
9	0.95	0.91 − 0.99	0.014	0.95	0.91 − 0.99	0.018	0.95	0.91 − 0.99	0.018
10	0.94	0.90 − 0.98	0.002	0.93	0.89 − 0.97	0.001	0.93	0.89 − 0.97	0.002
11	0.92	0.88 − 0.96	< 0.001	0.91	0.88 − 0.96	< 0.001	0.91	0.88 − 0.96	< 0.001
Ethnicity (White)									
Mixed	0.99	0.92–1.06	0.720	1.01	0.93–1.09	0.860	1.01	0.93–1.09	0.857
Asian	0.63	0.59 − 0.67	< 0.001	0.59	0.55 − 0.63	< 0.001	0.59	0.55 − 0.63	< 0.001
Black	0.83	0.76 − 0.91	< 0.001	0.79	0.55 − 0.63	< 0.001	0.79	0.72 − 0.88	< 0.001
Other	0.82	0.75 − 0.89	< 0.001	0.79	0.72 − 0.86	< 0.001	0.79	0.72 − 0.86	< 0.001
School level									
Socioeconomic status (Low)									
Medium	-	-	-	1.57	1.19–2.09	0.002	1.58	1.19–2.09	0.001
High	-	-	-	1.98	1.47–2.68	< 0.001	2.04	1.51–2.75	< 0.001
Rurality (Urban)									
Rural	-	-	-	0.66	0.50 − 0.85	0.002	0.68	0.52 − 0.87	0.003
Number of policies	-	-	-	1.06	1.00–1.14	0.091	-	-	-
Policy contents									
Promotional	-	-	-	-	-	-	1.15	0.92–1.45	0.228
Infrastructure	-	-	-	-	-	-	1.28	0.94–1.73	0.112
Training	-	-	-	-	-	-	0.70	0.49 − 0.99	0.049
Partnerships	-	-	-	-	-	-	1.23	0.96–1.56	0.097
Variance (Level 2)	0.78	0.64 − 0.95	-	0.61	0.49 − 0.75	-	0.58	0.47 − 0.71	-
Log likelihood	-66229.31			-59848.31			-59843.60		
AIC/BIC	132482.6 / 132598.6			119728.6 / 119881.6			119725.2 / 119906.9		
ICC – Constant only	0.19								
ICC (Level 1)	0.19								
ICC (Level 1 & 2)	0.15								

*N* = 36,361; *OR* = odds ratio; *ICC* = intra-class correlations. Level 1 refers to pupil-level variables and Level 2 refers to school-level variables. Model 1a includes pupil-level predictors only. Model 1b additionally includes school-level predictors, with a focus on the number of policies. Model 1c includes school-level predictors, focusing on policy content

In contrast, among secondary aged pupils (see Model 2b in Table 5), girls were less likely to report ATS compared to boys (OR = 0.82, 95%CI: 0.80–0.84) and pupils across years 8 to 11 had lower odds of active travel in comparison to year 7 pupils. Compared with pupils from a White background, those from an Asian (OR = 0.59, 95%CI: 0.55–0.63), Black or other ethnicity were less likely to report active travel. 15% of the variance in the likelihood of ATS was attributed to differences between schools, while the remaining 84% due to individual-level factors or residual variation.

### Associations between school-level policies and active travel rates

Among both primary and secondary schools, there was a clear gradient between school socioeconomic position and pupil active travel. That is (see Model 1b in Table 4, and Model 2b in Table 5), pupils from a medium (primary school: OR = 1.18, 95%CI: 1.00-1.39 and secondary school: OR = 1.57, 95%CI: 1.19–2.09) or high (primary school: OR = 2.22, 95%CI: 1.87–2.64 and secondary school: OR = 1.98, 95%CI: 1.47–2.68) socioeconomic school were more likely to report ATS compared to peers from a low socioeconomic school.

The odds of ATS were lower among pupils attending rural primary schools (OR = 0.71, 95%CI: 0.61–0.83) and rural secondary schools (OR = 0.66, 95%CI: 0.50–0.85), compared to peers in urban school settings.

**Fig. 1:**
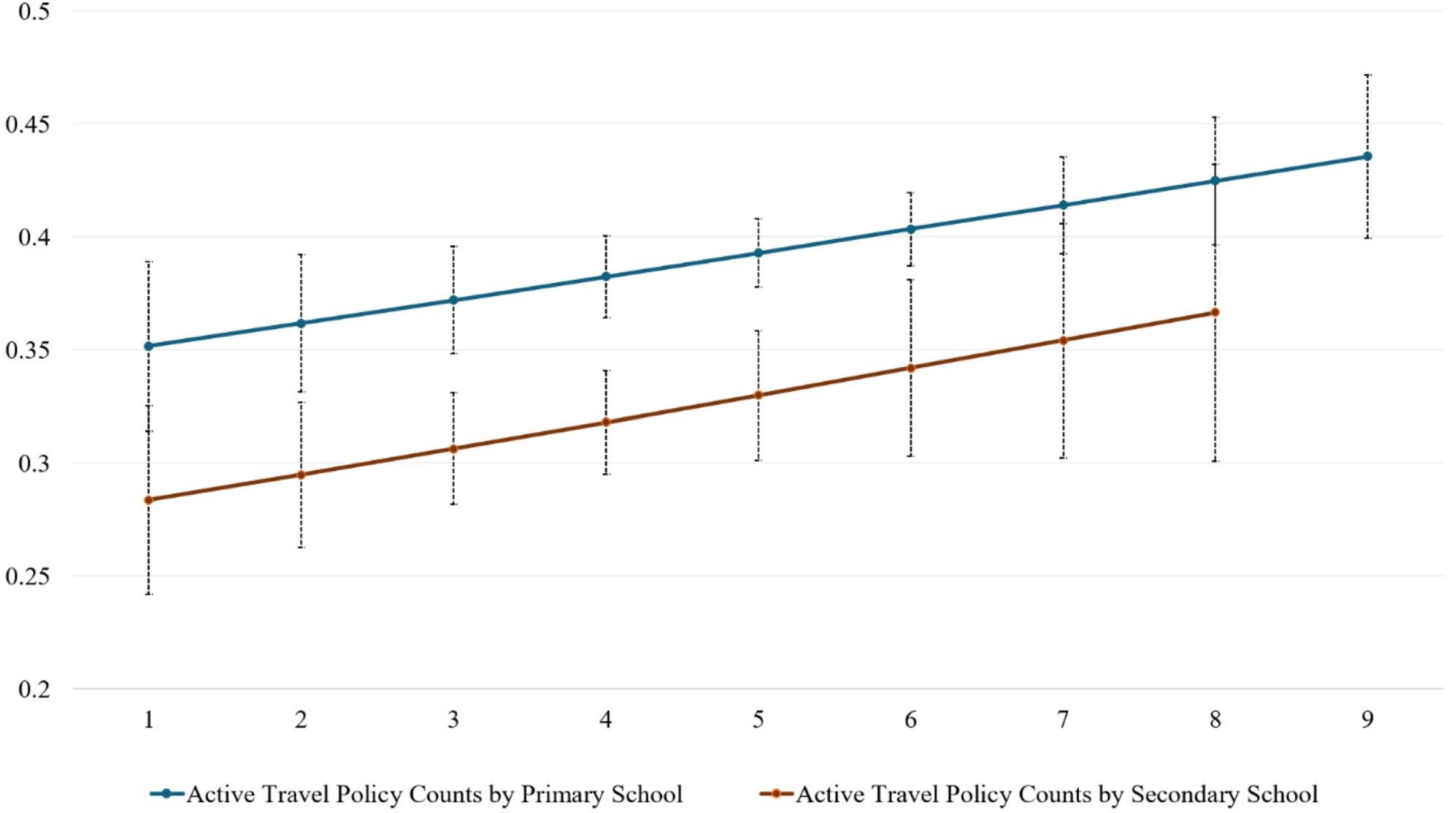
Predicted probability of student active travel with confidence interval by school travel policy

Figure 1 indicates that in schools with greater numbers of active travel policies, pupils tend to report higher levels of ATS. The multilevel model indicated that, among primary school pupils, each additional school policy was associated with a 5% increase in the odds of ATS (OR = 1.05, 95%CI 1.01–1.09). A similar trend was observed among secondary school pupils; however, the association was not statistically significant (OR = 1.06, 95%CI 0.99–1.14).

When examining associations between the different types of active travel school policies and reported pupil ATS rates, no associations were shown among 7-11-year-olds (Model 1c in Table 4). Among secondary schools, promotion (OR = 1.15, 95% CI: 0.92–1.45), infrastructure (OR = 1.28, 95% CI: 0.94–1.73), and partnerships (OR = 1.23, 95% CI: 0.96–1.56) were all positively associated with active travel to school, though none reached statistical significance (Model 2c in Table 5). In contrast, training policies were associated with lower odds of active travel (OR = 0.70, 95% CI: 0.49–0.99, *p* = .049).

## Discussion

This study sought to determine the prevalence of ATS and examine the individual and school-level factors associated with reports among children in Wales, UK. Around four in ten primary school pupils and three in ten secondary school pupils reported active travel, broadly consistent with European studies [51, 52], yet lower than earlier estimates reported in England [53].

### Individual demographics and active travel

This study contributes to the evidence base by using a nationally representative sample with multiple demographic indicators, as well as specifically examining differences of active travel rates among various ethnic groups. The present study found age and gender disparities in reported active travel, with gaps emerging during adolescence. In line with wider evidence on social norms [18, 52, 54], rates of active travel among 11-16-year-olds were lower among girls compared to boys. This echoes the broader physical activity landscape, with lower levels of overall physical activity, active leisure time and engagement in sport among girls [55].

Ethnic differences in ATS varied across school settings and developmental stages. Among 7–11-year-olds, Black pupils reported the highest prevalence, while Gypsy, Roma, or Traveller pupils reported the lowest. These patterns differ from a London-based study [21], which found lower ATS odds among Black pupils compared with White and other ethnic minority peers. Among 11–16-year-olds in the present study, pupils identifying as Black, mixed, or other ethnicities initially showed higher ATS rates than White peers, but adjusted models revealed a reversal, suggesting that contextual factors, such as urbanicity, school location, and socioeconomic status become more influential during adolescence. Taken together, these findings underscore the importance of considering age-specific contexts when examining ethnic differences in active travel. Future research should explore how these factors intersect and evolve to better understand opportunities and barriers across developmental stages.

### School characteristics and ATS

Contrary to some previous research suggesting that lower- socioeconomic status households exhibit higher rates of ATS [22, 23, 56], our findings revealed that pupils attending high-socioeconomic schools were approximately twice as likely to report ATS compared with those in low-socioeconomic settings. It is important to note that our analysis focused on school-level socioeconomic status (percentage of pupils eligible for free school meals) rather than individual household socioeconomic status. This distinction may partly explain the divergence from earlier studies, as school-level socioeconomic status can reflect neighbourhood characteristics, infrastructure quality, and prevailing social norms that influence active travel beyond individual family resources [57]. Interestingly, some studies examining household SES have reported similar patterns to ours, suggesting that higher-SES families may prioritise health and sustainability or reside in areas with safer walking environments [24, 25]. These nuances highlight the need for multi-level intervention strategies, as conceptualized by the Socio-ecological Model [10], to inform policy efforts that address individual behaviours, school-level contexts, and broader environmental conditions to promote equitable active school transport.

Our findings of lower ATS rates among pupils attending rural schools may reflect multiple factors. Infrastructure limitations, such as lack of connected footpaths or safe cycling routes and larger school catchment areas, are likely contributors. In addition, pupils in rural areas often face longer travel distances and reduced transport alternatives compared to those in urban settings, which may further constrain opportunities for active travel. Previous research emphasises that infrastructure quality, including segregated cycle lanes and coherent pedestrian networks, plays a critical role in enabling active travel and shaping parental perceptions of safety [58]. International evidence similarly indicates that rurality presents structural barriers to ATS, including dispersed housing patterns and limited route connectivity, which reduce the feasibility of walking or cycling to school [59]. Together, these findings point to potential targets for intervention efforts: addressing infrastructure gaps in rural areas and tailoring active travel strategies to the distinct barriers and resources associated with different socioeconomic contexts.

### School-level support and active travel policies

No significant differences emerged based on the presence of a single travel policy, yet our results suggest that implementing multiple, combined ATS policies may offer greater benefits, particularly within primary school settings. The multilevel analysis indicated that each additional school policy was associated with a 5% increase in the odds of ATS among primary school pupils, although this effect was modest. A similar trend was observed in secondary schools, but the association did not reach statistical significance, suggesting that policy impact may diminish as pupils age and gain greater autonomy over travel choices. These findings are consistent with emerging international evidence [60] which highlights that multi-component, whole‑school approaches tend to be more effective than isolated measures, particularly in younger age groups. They also align with systematic reviews indicating that multi-component interventions, those combining education, encouragement, engineering, and policy, are more effective than isolated measures [35, 61]. It is also important to consider the extent to which these policies are implemented in practice. International evidence [60] emphasises that the effectiveness of school-based physical activity and travel policies depends not only on their presence but on the consistency and quality of their implementation.

When examining specific policy types in the current study, no associations were observed among primary schools, whereas secondary schools showed positive, though non-significant associations for promotion, infrastructure, and partnerships. Interestingly, training policies were linked to lower odds of active travel, a finding that warrants further exploration. Similar patterns have been reported elsewhere, where safety-focused training may inadvertently heighten parental concerns, reducing independent mobility [62]. This finding also reveals a stark contrast in policy implementation: while most primary schools (76.13%) offer cycling proficiency training, such initiatives are rare in secondary settings (11.92%). This discrepancy may reflect perceptions that training is developmentally appropriate for younger children, whereas older pupils are considered less in need of such support. Anecdotal evidence from Welsh local authority active travel officers suggests that practical barriers, such as budget constraints and challenges in engaging secondary schools, also play a role. Furthermore, the two major organisations supporting schools to promote active travel (Sustrans, now Walk, Wheel, Cycle Trust, and Living Streets) primarily focus their efforts on primary schools. For example, many primary schools participate in Living Streets’ WOW Walk to School Challenge [63], which is rarely extended to secondary settings. These mixed results underscore the importance of integrating school-level policies with environmental improvements and community engagement, as recommended by Safe Routes to School frameworks [64]. Policy effectiveness is often contingent on contextual tailoring and implementation quality, reinforcing the need for comprehensive strategies that address both behavioural and environmental barriers [65].

The overall patterns observed in this study suggest that different age groups may face distinct barriers and facilitators to active travel. For younger pupils, associations suggest that efforts address cultural and structural barriers that persist across ethnic groups, while for adolescents, interventions could focus on broader environmental and socioeconomic factors. Evidence from the wider active‑travel literature indicates that neighbourhood safety, street connectivity, and travel distance are particularly important for supporting ATS during adolescence [59, 60]. Schools can play a pivotal role by adopting integrated active travel policies that combine infrastructure, education, and community engagement. In addition, strategies must consider the unique challenges of promoting ATS in rural schools, where larger catchment areas, limited transport alternatives, and infrastructure gaps require context-specific solutions.

### Strengths and limitations

Using a large, nationally representative sample of 7–16-year-olds in mainstream schools across Wales, this study provides robust population-level estimates for Wales, alongside generalisable evidence on how socioeconomic and school-contextual factors relate to active travel. Findings may inform work in other UK nations and internationally. It also provides a strong platform for future cross-national comparisons across differing policy environments.

Several limitations should be noted. First, reliance on self-reported data from pupils and school leaders introduces potential social desirability and recall bias. Second, ATS was measured based on pupils reported main mode of travel. This approach does not capture mixed-mode journeys, such as pupils who walk part of the way before using a non-active mode (e.g., car or bus). Additionally, pupils selecting ‘public transport’ may still accumulate some walking to and from stops or stations; as a result, this category may include a modest amount of physical activity and therefore sits between purely motorised and purely active modes. Consequently, ATS prevalence may be underestimated, and associations with school-level policies may not fully reflect partial active travel behaviours. Third, as a cross-sectional study, causal relationships cannot be inferred. Data collection occurred in the Autumn term, so seasonality may have influenced active travel rates. Additionally, the focus on journeys to school excludes return trips, likely underestimating prevalence. Fourth, although individual‑level SES was collected via the Family Affluence Scale (FAS), it could not be used in analyses due to non‑comparable versions across primary and secondary settings and substantial missingness among primary school pupils. As a result, SES was operationalised at the school level, which limits our ability to distinguish compositional from contextual effects. Finally, a further limitation is the absence of information on pupils’ distance or travel time to school. Distance is one of the strongest predictors of active school travel, and without this information our analyses cannot fully account for geographical or catchment-related constraints. As a result, socioeconomic or environmental patterns should be interpreted cautiously, as these may partly reflect unmeasured variation in travel distances.

## Conclusions

Active travel behaviours among primary and secondary school pupils are shaped by individual, school, and environmental factors, with meaningful shifts most likely when supportive conditions exist. Pupils facing the greatest barriers may benefit most from targeted school initiatives, although further research is needed. As the first population-level analysis linking pupil and school data on active travel in Wales, this study offers evidence that may help inform policymakers seeking equitable, context-sensitive approaches to school-based physical activity promotion.

These findings come at a critical time, given concerns about future funding for active travel and ambitions set out in the Regional Transport Plans [66]. The patterns observed align with national health and transport strategies, which call for integrated whole system approaches. While grounded in the Welsh context, the results have wider relevance for regions and countries aiming to embed active travel within school and community frameworks. Structural actions, such as improving infrastructure, tailoring interventions to developmental stages, and addressing equity concerns, appear important for supporting active travel. and reduce health disparities globally.

## Supplementary Information

Supplementary Material 1.

## Data Availability

Data used in this study were obtained from The School Health Research Network (SHRN) at Cardiff University, UK. Data collected via The SHRN Student Health and Well-being Survey are available for research purposes upon completion and approval of a data access request. Please contact [shrn@cardiff.ac.uk](mailto: shrn@cardiff.ac.uk) for further information.

## Footnotes

1 Maintained primary and secondary schools are state-funded institutions overseen by local authorities that follow the national curriculum and provide free education to students aged 5 to 16 across Wales and England.

2 The term “Gypsy, Roma or Traveller” follows the Welsh Government’s consolidated definition, which includes persons with a nomadic way of life (present or past), Romani Gypsies, Irish Travellers, Travelling Showpeople, and others with a recognised cultural tradition of nomadism.

3 PCSOs are uniformed police staff in Wales and England who provide a visible, approachable presence in communities. They support community policing and help address local safety and antisocial behaviour issues.

## References

[1] Campos-Garzón P, et al. Contribution of active commuting to and from school to device-measured physical activity levels in young people: A systematic review and meta-analysis. Scand J Med Sci Sports. 2023;33(11):2110–24. 37497601 10.1111/sms.14450

[2] Organization WH. *COP24 special report: health and climate change.* 2018.

[3] Barros P, et al. Chapter Four - Impact of active travel to school on children’s health: an overview of systematic reviews. In: Mindell JS, Watkins SJ, editors. Advances in Transport Policy and Planning. Editors: Academic; 2024. pp. 145–65.

[4] Heelan KA, et al. Evaluation of a walking school bus for promoting physical activity in youth. J Phys Act Health. 2009;6(5):560–7. 19953832 10.1123/jpah.6.5.560

[5] Lubans DR, et al. The relationship between active travel to school and health-related fitness in children and adolescents: a systematic review. Int J Behav Nutr Phys Activity. 2011;8(1):5. 10.1186/1479-5868-8-5 PMC3039551 21269514

[6] Villa-González E, et al. Effects of a school-based intervention on active commuting to school and health-related fitness. BMC Public Health. 2017;17(1):20. 28056914 10.1186/s12889-016-3934-8 PMC5216538

[7] Waygood E, et al. Transport and child well-being: An integrative review. Travel Behav Soc. 2017;9:32–49.

[8] Stark J, Singleton PA, Uhlmann T. Exploring children’s school travel, psychological well-being, and travel-related attitudes: Evidence from primary and secondary school children in Vienna, Austria. Travel Behav Soc. 2019;16:118–30.

[9] Ding D, et al. The co-benefits of active travel interventions beyond physical activity: a systematic review. Lancet Planet Health. 2024;8(10):e790–803. 39393380 10.1016/S2542-5196(24)00201-8

[10] Bronfenbrenner U. *Ecological Systems Theory. In: U. Bronfenbrenner, editor, Making human beings human: Bioecological perspectives on human development). Thousand Oaks, CA: Sage Publications Ltd*. 2005: 106–173.

[11] Klos L, et al. Active school transport routines during school transitions: Socio-structural predictors of changes from childhood into early adulthood. Volume 81. Health & Place; 2023:103005. 10.1016/j.healthplace.2023.103005 37003019

[12] Shaw B et al. *Children’s independent mobility: an international comparison and recommendations for action.* 2015.

[13] Government W. *National Survey for Wales 2021-22. Technical Report. 2022.* https://www.gov.wales/sites/default/files/statistics-and-research/2022-07/national-survey-for-wales-april-2021-to-march-2022-technical-report.pdf *Accessed 13 June* 2023.

[14] Wilson OW, et al. Results from Aotearoa New Zealand’s 2022 Report Card on Physical Activity for Children and Youth: A call to address inequities in health-promoting activities. J Exerc Sci Fit. 2023;21(1):58–66. 36408209 10.1016/j.jesf.2022.10.009 PMC9663885

[15] Smith M, et al. Trends and measurement issues for active transportation in New Zealand’s physical activity report cards for children and youth. J Transp Health. 2019;15:100789.

[16] Ikeda E, et al. Built environment associates of active school travel in New Zealand children and youth: A systematic meta-analysis using individual participant data. J Transp Health. 2018;9:117–31.

[17] Boland P, Nowland R, Tellis KD, Adams M, Westwood J, Crook D, Larkins C, Ridley J. Barriers and Facilitators to Cycling to School for Children in the UK: A Systematic Review. Act Travel Stud, 2025; 5(1).

[18] Goel R, et al. Gender differences in active travel in major cities across the world. Transportation. 2023;50(2):733–49. 37035250 10.1007/s11116-021-10259-4 PMC7614415

[19] Sustrans. *UK Report: The Children’s Walking and Cycling Index*. 2025.

[20] Pouliou T, Fry R, Keshetty R, Pedrick-Case R, Johnson R, Lyons J, et al. Active travel to school for children in Wales: A data linkage project and exploratory analysis. ADR Wales; 2025.

[21] Bosch L, et al. Associations of the objective built environment along the route to school with children’s modes of commuting: A multilevel modelling analysis (the SLIC study). PLoS ONE. 2020;15(4):e0231478. 32271830 10.1371/journal.pone.0231478 PMC7145202

[22] Laverty AA, et al. Associations of active travel with adiposity among children and socioeconomic differentials: a longitudinal study. BMJ Open. 2021;11(1):e036041. 33436461 10.1136/bmjopen-2019-036041 PMC7805367

[23] Noonan RJ. Family income matters! Tracking of habitual car use for school journeys and associations with overweight/obesity in UK youth. J Transp Health. 2021;20:100979.

[24] Kobel S, Wartha O, Steinacker J. Correlates of active transport to school in German primary school children. Dtsch Z Sportmed. 2019;2019:67–74.

[25] Wex I, et al. Active school transport in an urban environment:prevalence and perceived barriers. BMC Public Health. 2023;23(1):557. 36959624 10.1186/s12889-023-15464-7 PMC10037850

[26] Timperio A, et al. Associations between parental perceptions of neighbourhood environments and active travel to school: IPEN Adolescent study. Int J Behav Nutr Phys Activity. 2025;22(1):55. 10.1186/s12966-025-01738-3 PMC12079927 40375328

[27] Prince SA, et al. Examining the state, quality and strength of the evidence in the research on built environments and physical activity among children and youth: An overview of reviews from high income countries. Health Place. 2022;76:102828. 35700605 10.1016/j.healthplace.2022.102828

[28] Ikeda E, et al. Assessment of direct and indirect associations between children active school travel and environmental, household and child factors using structural equation modelling. Int J Behav Nutr Phys Activity. 2019;16(1):32. 10.1186/s12966-019-0794-5 PMC6451289 30953526

[29] Pulimeno M, et al. School as ideal setting to promote health and wellbeing among young people. Health Promot Perspect. 2020;10(4):316–24. 33312927 10.34172/hpp.2020.50 PMC7723000

[30] WorldHealth Organisation. *Global action plan on physical activity 2018–2030: more active people for a healthier world. 2018.* https://www.un.org/development/desa/dspd/wp-content/uploads/sites/22/2019/09/WHO_GAPPA_2018-2030.pdf. *Accessed 21 August* 2019.

[31] Panel ER. *Cross Party Group on the Active Travel Act: Review of the Active Travel (Wales) Act 2013*. 2022.

[32] Government W. *Active Travel Act Guidance July* 2021. https://www.gov.wales/sites/default/files/publications/2022-01/active-travel-act-guidance.pdf. *Accessed 01 February 2022*.

[33] Welsh Government. *Active Travel Board annual reports.* https://www.gov.wales/active-travel-board-annual-reports*. Accessed 21 August* 2025.

[34] Wales A. *Active Travel Report 2024. *https://www.audit.wales/publication/active-travel*. Accessed 21 May* 2025. 2024.

[35] Larouche R, et al. Effectiveness of active school transport interventions: a systematic review and update. BMC Public Health. 2018;18(1):206. 29390988 10.1186/s12889-017-5005-1 PMC5796594

[36] Roaf E, Larrington-Spencer H, Lawlor ER. Interventions to increase active travel: A systematic review. J Transp Health. 2024;38:101860.

[37] Stark J, Berger WJ, Hössinger R. The effectiveness of an intervention to promote active travel modes in early adolescence. Transp Res part F: traffic Psychol Behav. 2018;55:389–402.

[38] Aranda-Balboa MJ, et al. The effect of a school-based intervention on children’s cycling knowledge, mode of commuting and perceived barriers: a randomized controlled trial. Int J Environ Res Public Health. 2022;19(15):9626. 35954982 10.3390/ijerph19159626 PMC9367827

[39] Humberto M, Moura F, Giannotti M. Can outdoor activities and inquiry sessions change the travel behavior of children and their caregivers? Empirical research in public preschools in São Paulo (Brazil). J Transp Geogr. 2021;90:102922.

[40] Rothman L, et al. Pilot study to evaluate school safety zone built environment interventions. Inj Prev. 2022;28(3):243–8. 34462331 10.1136/injuryprev-2021-044299 PMC9132849

[41] Lambe B, Murphy N, Bauman A. Active travel to primary schools in Ireland: an opportunistic evaluation of a natural experiment. J Phys Activity Health. 2017;14(6):448–54. 10.1123/jpah.2016-0429 28253069

[42] Smith M, et al. Impact of changing road infrastructure on children’s active travel: a multi-methods study from Auckland, New Zealand. J Transp Health. 2020;18:100868.

[43] Mendoza JA, et al. Bicycle trains, cycling, and physical activity: a pilot cluster RCT. Am J Prev Med. 2017;53(4):481–9. 28668251 10.1016/j.amepre.2017.05.001 PMC5894119

[44] Pérez-Martín P, et al. Evaluation of a walking school bus service as an intervention for a modal shift at a primary school in Spain. Transp Policy. 2018;64:1–9.

[45] Biondi B, Romanowska A, Birr K. Impact evaluation of a cycling promotion campaign using daily bicycle counters data: The case of Cycling May in Poland. Transp Res part A: Policy Pract. 2022;164:337–51.

[46] Coombes E, Jones A. Gamification of active travel to school: A pilot evaluation of the Beat the Street physical activity intervention. Health Place. 2016;39:62–9. 26974232 10.1016/j.healthplace.2016.03.001 PMC5405045

[47] Murphy S, et al. A Transdisciplinary Complex Adaptive Systems (T-CAS) Approach to Developing a National School-Based Culture of Prevention for Health Improvement: the School Health Research Network (SHRN) in Wales. Prev Sci. 2021;22(1):50–61. 30536190 10.1007/s11121-018-0969-3 PMC7762741

[48] Page N, Liu S, Morgan K, Angel L et al. Data Resource Profile: The School Health Research Network (SHRN) Student Health and Well-being (SHW) survey of 11–16-year-olds (2017–2023). Int J Epidemiol, 2024; 53(6). 10.1093/ije/dyae161 PMC11645470 39657065

[49] Currie C, Gabhainn N, Godeau E. The Health Behaviour in School-aged Children: WHO Collaborative Cross-National (HBSC) Study: origins, concept, history and development 1982–2008. Int J Public Health. 2009;54(2):131–9. 19639260 10.1007/s00038-009-5404-x

[50] Wales S. *Pupil Level Annual School Census (PLASC) and Office for National Statistics rural/urban classification*. 2022.

[51] Schicketanz J, et al. On foot or by car: what determines children’s active school travel? Children’s Geographies. 2022;20(2):174–88.

[52] Brindley C, et al. Gender-specific social and environmental correlates of active travel to school in four European countries: the HBSC Study. Front Public Health. 2023;11:1190045. 37559734 10.3389/fpubh.2023.1190045 PMC10407096

[53] Department for Transport. *National Travel Survey 2022: Travel to and from school. 2023. Accessed at*: https://www.gov.uk/government/statistics/national-travel-survey-2022/national-travel-survey-2022-travel-to-and-from-school*on 03 April 2024*.

[54] Levi S, et al. Adolescent active travel and physical activity: Role of social media, norms and the environment. J Transp Health. 2024;36:101796.

[55] Duffey K et al. Barriers and Facilitators of Physical Activity Participation in Adolescent Girls: A Systematic Review of Systematic Reviews. Front Public Health, 2021: 9. 10.3389/fpubh.2021.743935 PMC8553996 34722450

[56] Panter JR, et al. Neighborhood, route, and school environments and children’s active commuting. Am J Prev Med. 2010;38(3):268–78. 20171528 10.1016/j.amepre.2009.10.040 PMC3819023

[57] Tan CY, et al. Meta-analytical insights on school SES effects. Educational Rev. 2025;77(1):274–302.

[58] Mitra R. Independent mobility and mode choice for school transportation: a review and framework for future research. Transp reviews. 2013;33(1):21–43.

[59] White B, et al. Comparison of physical activity patterns across large, medium and small urban areas and rural settings in the Otago Region, New Zealand. N Z Med J. 2021;134(1534):51–65. 33927438

[60] Kuhn AP, et al. Student perceptions of U.S. based school day physical activity best practices in relation to accelerometer-based sedentary behavior and activity. Prev Med Rep. 2025;49:102944. 39807184 10.1016/j.pmedr.2024.102944 PMC11729001

[61] Buttazzoni AN, et al. Active school travel intervention methodologies in North America: a systematic review. Am J Prev Med. 2018;55(1):115–24. 29776785 10.1016/j.amepre.2018.04.007

[62] Eyler AA, et al. Policies related to active transport to and from school: a multisite case study. Health Educ Res. 2008;23(6):963–75. 17956883 10.1093/her/cym061

[63] Living Streets. WOW - the walk to school challenge. 2025. https://www.livingstreets.org.uk/walk-to-school/primary-schools/wow-the-walk-to-school-challenge/*. Accessed 21 September 2025*.

[64] Yuan M, Ermagun A. A systematic review of the safe routes to school program: A 10-principle policy effectiveness framework for future investments. Transp Policy, 2025: 103846.

[65] Ikeda E, et al. Keeping kids safe for active travel to school: A mixed method examination of school policies and practices and children’s school travel behaviour. Travel Behav Soc. 2020;21:57–68. 33014711 10.1016/j.tbs.2020.05.008 PMC7473447

[66] Welsh Government. *Regional transport plans: improving travel in your area.* https://www.gov.wales/regional-transport-plans-improving-travel-your-area*. Accessed 21 July* 2025. 2025.

