# Supplementary material for: Policy on the move: active travel to school among 7–16-year-olds in Wales and its links to school policy and socio-demographic factors
Source: BMC Public Health. 2026 Mar 13;26:1298. doi: 10.1186/s12889-026-26995-0 (PMC13097712; doi:10.1186/s12889-026-26995-0)
Supplement: Supplementary file 1 — Supplementary Material 1. [file 12889_2026_26995_MOESM1_ESM.docx]

**Supplementary File 1**

**Table A.** *Missing Observations for Variables among Primary school data*

| **Variable** | **Available Obs.** | **Missing Obs.** | **% Missing** |
| --- | --- | --- | --- |
| Active travel policy | 44,100 | 3,781 | 7.90% |
| FSM | 41,071 | 6,810 | 14.22% |
| School location | 44,068 | 3,813 | 7.96% |
| Grade | 47,840 | 41 | 0.09% |
| Gender | 47,024 | 857 | 1.79% |
| Family Affluence Scale | 34,597 | 13,284 | 27.74% |
| Ethnicity | 40,235 | 7,646 | 15.97% |
| Pupil active travel | 45,288 | 2,593 | 5.42% |

**Table B.** *Missing Observations for Variables among Secondary school data*

| **Variable** | **Available Obs.** | **Missing Obs.** | **% Missing** |
| --- | --- | --- | --- |
| Active travel policy | 115,275 | 10,778 | 8.55% |
| FSM | 125,049 | 1004 | 0.80% |
| School location | 125,731 | 322 | 0.26% |
| Grade | 126,043 | 10 | 0.01% |
| Gender | 125,016 | 1037 | 0.82% |
| Family Affluence Scale | 118,364 | 7689 | 6.10% |
| Ethnicity | 119,964 | 6089 | 4.83% |
| Pupil active travel | 122,332 | 3721 | 2.95% |

**Table C.** *Distribution of Participating and Non-Participating Primary Schools Across Welsh Local Health Boards (LHBs)*

| **Health Board** | **Non-Participating (%)**  **(%)** | **Participating (%)** | **Total** |
| --- | --- | --- | --- |
| Aneurin Bevan | 56 (29.8%) | 132 (70.2%) | 188 |
| Betsi Cadwaladr | 264 (80.7%) | 63 (19.3%) | 327 |
| Cardiff & Vale | 74 (50.7%) | 72 (49.3%) | 146 |
| Cwm Taf Morgannwg | 74 (46.0%) | 87 (54.0%) | 161 |
| Hywel Dda | 107 (56.9%) | 81 (43.1%) | 188 |
| Powys | 44 (62.9%) | 26 (37.1%) | 70 |
| Swansea Bay | 82 (62.6%) | 49 (37.4%) | 131 |
| Total | 701 (57.9%) | 510 (42.1%) | 1,211 (100%) |

***Note****.* Data is aggregated by LHB-level to align with the seven strategic administrative units responsible for healthcare delivery and policy implementation across Wales.

**Table D.** *Distribution of Participating and Non-Participating Primary Schools Based on Urban/Rural Location*

| **Rural/Urban** | **Non-Participating (%)** | **Participating (%)** | **Total** |
| --- | --- | --- | --- |
| Rural | 352 (66.4%) | 178 (33.6%) | 530 (100%) |
| Urban | 349 (51.6%) | 328 (48.5%) | 677 (100%) |
| Missing | 0 | 4 (100.0%) | 4 (100%) |
| Total | 701 (57.9%) | 510 (42.1%) | 1,211 (100%) |

**Table E.** *Distribution of Participating and Non-Participating Primary Schools Based on Free School Meal (FSM).*

| **FSM** | **Non-Participating School** | **Participating School** |
| --- | --- | --- |
| Low FSM | 204 (31.58%) | 159 (33.76%) |
| Medium FSM | 224 (34.67%) | 157 (33.33%) |
| High FSM | 218 (33.75%) | 155 (32.91%) |
| Total | 646 (100%) | 471 (100%) |

***Note:*** FSM cannot be linked to some schools because the relevant data are missing from the Welsh Government (n.d.) administrative records. As a result, the total number of schools is lower than in the previous tables.

**Reference**

Welsh Government. (n.d.). *Pupil Level Annual School Census summary data by school (pupils aged 5 to 15 in primary, middle or secondary schools)* [Data set]. StatsWales. Retrieved March 3, 2026, from <https://statswales.gov.wales/Catalogue/Education-and-Skills/Schools-and-Teachers/Schools-Census/Pupil-Level-Annual-School-Census/pupillevelannualschoolcensussummarydata-by-school>
